# The Big Five and Big Two personality factors in Mongolia

**DOI:** 10.3389/fpsyg.2022.917505

**Published:** 2022-07-26

**Authors:** Michael Minkov, Boris Sokolov, Marc Albert Tasse, Erdenebileg Jamballuu, Michael Schachner, Anneli Kaasa

**Affiliations:** ^1^Varna University of Management, Sofia, Bulgaria; ^2^University of Tartu, Tartu, Estonia; ^3^Ronald Inglehart Laboratory for Comparative Social Research, National Research University Higher School of Economics, Moscow, Russia; ^4^SICA, Ulaanbaatar, Mongolia; ^5^Vrije Universiteit, Amsterdam, Netherlands

**Keywords:** Big Five, Big Two, Mongolia, culture, personality

## Abstract

Etic psychometric tools work less well in non-Western than in Western cultures, whereas data collected online in the former societies tend to be of superior quality to those from face-to-face interviews. This represents a challenge to the study of the universality of models of personality and other constructs. If one wishes to uncover the true structure of personality in a non-Western nation, should one study only highly educated, cognitively sophisticated Internet users, and exclude the rest? We used a different approach. We adapted a short Big Five tool, previously tested successfully in 19 countries on all continents, to Mongolian culture. EFA and CFA analyses across a nationally representative sample of 1,500 adult Mongolians recovered the Big Five satisfactorily. A Big Two (plasticity and stability) model was also recovered reasonably well. Correlations between personality traits and age, as well as gender differences, were not different from those reported for Western samples. Respondents with higher education, or higher-than-average socioeconomic status, or urban dwellers, or Internet users, did not yield a clearer Big Five than the whole sample. Our method (tool adaptation to a local cultural context) may be preferable to exclusion of specific demographic groups in Big Five studies of non-Western populations.

## Introduction

The five-factor model of personality and its close variant, known as the Big Five, seems to replicate well in societies across the globe (McCrae, 2002), at least when the etic approach (rather than emic approaches) is used. A recent study using large and nearly nationally representative samples from 19 countries on all continents revealed that a short Big Five tool, based on the BFI and other instruments tested in previous studies, achieves good configural and acceptable metric (though not full scalar) equivalence in all of them (Minkov et al., 2019). However, that study also found that a measure of the reliability of the tool in each nation was strongly correlated with national educational achievement. Laajaj et al. (2019) report that Big Five tools work less well in nations that are not WEIRD (Western, Educated, Industrialized, Rich, and Democratic) (Henrich et al., 2010), especially when the data are collected in face-to-face interviews rather than online. Online respondents in those countries may be more cognitively sophisticated and therefore more capable of providing coherent responses to questionnaire items. This means that the replicability of the Big Five may depend on socioeconomic factors, including education. This phenomenon is not a peculiarity of the Big Five. It seems to be a general characteristic of psychometric tools. For instance, Fontaine et al. (2008) found that Schwartz’s model of values replicates less well in societies at a low level of socioeconomic development. Vignoles et al. (2016) report the highest factor loadings on their measures of selfhood for “West” and the lowest for Sub-Saharan Africa. Schmitt and Allik’s (2005) administration of the Rosenberg self-esteem scale in 53 nations demonstrates that the strength of the correlation between all negatively and all positively worded items (which should be high and positive) is strongly correlated with a nation’s wealth per person and educational achievement.

We note that the six-factor model of personality, known as HEXACO, has been found to exhibit cross-cultural properties similar to those of the Big Five. In comparative multi-country analyses, HEXACO achieves acceptable configural and metric invariance though not scalar invariance (Thielmann et al., 2020; García et al., 2022). However, we focused our study on the Big Five for two reasons. First, these recent HEXACO studies were not available at the time of the conceptualization or our study, and we did not possess enough information concerning the cross-cultural recoverability of the HEXACO model. Also, publications such as Laajaj et al. (2019) and Minkov et al. (2019) provide clues as to why specifically the Big Five model may not be recovered sufficiently well in non-WEIRD countries but give no clues about HECAXO. We were particularly interested in the finding that the type of data collection and the education of the respondents may affect the recoverability of the Big Five model.

Cemalcilar et al. (2021) note that scholars call for testing Big Five inventories in non-WEIRD samples so as to minimize suspicions of bias in conclusions from Big Five studies. We answer that call in this study. We tested a Big Five tool across a nationally representative sample of Mongolia, a country where neither personality nor culture has been studied previously with psychometric tools.

### Research questions

We were interested in the following questions:

1. Is the Big Five model recoverable from our Mongolian data?
2. Do some groups of respondents, for instance, those with higher education, or higher socioeconomic status, or urban rather than rural, or online rather than face-to-face, yield clearer pictures of the Big Five in comparison to the whole, nationally representative sample?
3. If the Big Five is recoverable in Mongolia, does it have the hierarchical structure reported in studies of WEIRD samples? For instance, can we extract a Big Two (DeYoung, 2006) similar to that in WEIRD-based studies?
4. Do the various demographic groups of Mongolia exhibit Big Five differences as in studies of WEIRD samples? For instance, do extraversion and openness fall with age and does agreeableness rise (Donnellan and Lucas, 2008)? Do women have a higher mean score on neuroticism and agreeableness (South et al., 2018)?

## Materials and methods

Our data were collected by SICA, a Mongolian opinion poll agency, with a good track record of predicting election results, attesting to the reliability of its data collection methods. The sample consisted of 1,500 adult Mongolians selected proportionately from all inhabited places and all demographic groups of Mongolia so as to reflect the census of that country. Details are provided in Table 1.

**TABLE 1:** Sample characteristics.

Total number		1,500
Gender	Female	770 (51%)
	Male	730 (49%)
Age		Proportionately sampled within the 18–88 years range, with a mean of 41.34 and an SD of 16.42
Place of the Interview	Ulaanbaatar Note: Half of Mongolia’s population lives in Ulaanbaatar.	750
	Khentii	188
	Selenge	187
	Bayankhongor	187
	Uvs	188
Employment	Herder	372
	Self-employed	239
	Private sector	190
	Government	184
	Student	129
	Unemployed	110
	NGO	45
	International organization	8
Education	Special secondary	681
	Bachelor and above	483
	Secondary	196
	Vocational	80
	Primary	38
	No education	22

We used a tailor-made questionnaire, starting from the one by Minkov et al. (2019) in view of its good cross-cultural performance. Our strategy was to expand that questionnaire with more items so as to cover at least each of the 15 facets (three per factor) in Soto and John’s, (2017a,2017b) BFI-2 or the corresponding facets in other instruments provided by those authors (Table 1, p. 121). Those authors indicate that those 15 facets are the ones that tend to replicate across alternative hierarchical models and capture a large amount of personality information. We wrote six items per factor (eight for agreeableness and openness) so as to have a sufficiently large pool of items that could later be reduced to a short instrument by selecting best-performing items in terms of psychometric criteria, such as “item-total correlations” (Gosling et al., 2003). This approach was used to develop the earliest Big-Five instruments in the 1960’s (Goldberg, 1992). Our new items were based on existing scales, such as Goldberg’s (1992) adjectives. For instance, his extraversion markers “silent-talkative” and “unenergetic-energetic” (Table 1, p. 31) were used to generate the statements “I often talk a lot” versus “I usually keep quiet” and “I am a lively person full of energy” versus “I often seem to be slow or sleepy, without much energy.” The BFI and Konstabel et al.’s (2012) instrument, as well as Gosling et al.’s (2003) Big Five descriptors, were also taken into consideration. Some items were derived from literature definitions of Big Five traits. For example, the statement “I often feel sad” is based on Soto and John’s (2017b) explanation of neuroticism: “an individual’s general tendency to experience negative emotions such as anxiety and sadness” (p. 120). The final item selection was made after consultations with Mongolian social scientists on the items (genitive case) translatability, comprehensibility, and meaningfulness in the Mongolian cultural context. Brevity was also considered important. For instance, Konstabel et al.’s (2012) extraversion statement “I am optimistic and mostly in good spirits. Sometimes I am exuberantly happy” (Appendix, p. 28) was reduced to “I often laugh.”

A pitfall that we tried to avoid was the use of negatively worded items. Weems et al. (2006) found that such items can have an adverse effect on the ability of respondents with insufficient reading skills to provide adequate responses, whereas Roszkowski and Soven (2010) concluded that negatively worded items can produce a methodological artifact, probably due to carelessness. Schmitt et al.’s (2005) large cross-cultural study of self-esteem revealed that positively and negatively worded items on Rosenberg’s self-esteem scales, which were supposed to be highly and positively correlated, in fact yielded low and even insignificant correlations in some non-WEIRD countries.

We note that although our English-language questionnaire was based on previous publications, it was adapted and finalized with the participation of social scientists who speak English and Mongolian as native languages. For that reason, items that might be difficult to translate or comprehend in a Mongolian cultural context were avoided from the start, which greatly facilitated the subsequent translation process. Nevertheless, previous Big Five questionnaire translation practices in non-WEIRD countries were taken into account and followed, such as involvement of more than one translator (Piedmont and Chae, 1997; Kallasmaa et al., 2000; Thalmayer and Saucier, 2014; Hanif, 2018), avoidance of literal translation that may not sound well in the target language (Sergeeva et al., 2016), discussion of the translation by a group of researchers who speak the target language as a mother tongue (Kallasmaa et al., 2000; Sergeeva et al., 2016; Khan et al., 2019) including consideration of different versions of the same item (Kallasmaa et al., 2000), and back-translation into English (Thalmayer and Saucier, 2014; Hanif, 2018).

The wording of the items followed Minkov et al. (2019): a forced choice of two opposites plus a neutral “in-between” option. The whole questionnaire is provided in the Supplementary material.

Following Soto and John (2017b), we tested the structure that all our items would yield with an exploratory principal components analysis (PCA). Although the PCA recovered the Big Five, some items had low loadings on targeted factors. Retaining just the three highest-loading items per component, we produced a short 15-item Big Five instrument, as in Soto and John (2017a,b) and Minkov et al. (2019).

We are aware of Fokkema and Greiff’s (2017) objections to the common practice of performing a PCA and a CFA on the same dataset as that may result in overfitting. However, those authors admit that this danger decreases with the increase of a sample size. Our sample size is 1,500 and fully nationally representative, whereas they used an opportunity sample of 300.

Therefore, problematic overfitting is less likely in our case than in theirs.

We performed a confirmatory factor analysis (CFA) of the 15 items across all respondents and then across groups that may yield a clearer picture of the Big Five: only respondents with higher education, only urban dwellers, only online respondents, and only respondents whose monthly income is more than a million tugriks (about 300 EUR), which is roughly the income of the richest one-third of the sample. We also tested for measurement invariance (Steenkamp and Baumgartner, 1998) of the proposed model across several socio-demographic groups of the Mongolian population, including the four mentioned above, as well as female vs. male respondents.

In addition, we performed a PCA of the 15 items with oblique rotation and then factor-analyzed the oblique factors to ascertain if a Big Two emerged from the data: stability (low N, high C, and high A) and plasticity (high E and high O) (DeYoung, 2006). We also complemented that exploratory analysis by a hierarchical CFA model.

Next, we compared the Big Five scores of men and women and obtained correlations between Big Five scores and age. Gender and age are both based on biological realities, and if the Big Five model is universal the associations between gender, age, and Big Five traits in Mongolia should be similar to those known from studies in WEIRD countries.

## Results

After dropping items that did not load high on targeted factors, we obtained a five-factor solution, which, however, was characterized by some low loadings.

The solution is provided in Table 2.

**TABLE 2:** A principal component solution after dropping items that do not load on targeted factors.

	Component				

	1	2	3	4	5
O7	**0.704**	0.105	−0.023	0.058	−0.053
O5	**0.657**	0.148	0.009	0.141	−0.099
O6	**0.572**	0.178	−0.072	0.037	0.003
O4	**0.492**	−0.079	−0.034	−0.012	0.053
O8	**0.430**	0.193	0.042	−0.154	0.108
O1	**0.396**	0.041	0.090	−0.031	0.077
O3	**0.362**	−0.027	0.074	0.092	0.136
Ex3	−0.030	**0.727**	0.078	0.009	0.004
Ex6	0.155	**0.594**	0.001	−0.015	0.163
Ex4	0.080	**0.593**	0.163	−0.071	0.187
Ex5	0.208	**0.581**	−0.183	0.163	−0.024
Ex2	0.133	**0.296**	−0.191	0.058	−0.291
N5	−0.020	0.117	**0.650**	−0.058	−0.051
N1	0.038	0.173	**0.622**	−0.034	−0.039
N6	−0.046	−0.074	**0.559**	0.127	0.109
N4	0.129	−0.080	**0.554**	−0.167	−0.132
N3	0.061	−0.119	**0.484**	−0.255	0.020
Co1	−0.005	0.028	−0.055	**0.610**	−0.023
Co6	−0.015	0.051	0.003	**0.603**	−0.060
Co4	0.019	−0.041	−0.183	**0.518**	0.128
Co5	0.173	0.015	0.026	**0.509**	0.169
Co3	0.010	−0.010	−0.086	**0.457**	0.265
Ag3	0.035	0.013	−0.144	−0.133	**0.602**
Ag4	−0.003	−0.016	0.064	0.138	**0.597**
Ag1	0.027	0.133	−0.007	0.100	**0.546**
Ag2	0.275	0.043	0.054	0.116	**0.412**
Ag8	0.099	0.132	−0.073	0.148	**0.406**

*Bold values refer to item loadings on targeted factors.*

Next, we retained just the three highest-loading items on each component and repeated the PCA.

Table 3 shows the outcome of that analysis. The 15 remaining items produced five components with eigenvalues over 1.00, explaining 48.52% of variance. The item loadings are only slightly lower than those of the nationally representative US sample in Minkov et al. (2019). In Table 3, we indicate the trait facet that each item captures, whereas the whole wording of each item is provided in the Supplementary material. With a few exceptions, all facet terms were borrowed from Soto and John’s (2017b)Table 1, where they compare the terminology of different Big Five models. Evidently, the 15 items in Table 3 correspond to 15 Big Five facets or three for each of the five traits. This ensures as wide coverage of personality as is possible with a short 15-item tool.

**TABLE 3:** A principle components solution with the 15 highest-loading items on targeted factors.

Rotated component matrix					
	Component				
	1	2	3	4	5
Ex3 Activity	**0.747**	0.047	−0.012	0.012	−0.036
Ex6 Positive emotions	**0.656**	0.130	0.061	0.001	0.156
Ex4 Sociability/excitability	**0.645**	−0.029	0.141	0.011	0.076
N6 Insecurity	−0.151	**0.717**	0.000	0.098	0.128
N5 Volatility	0.108	**0.707**	−0.058	−0.115	−0.081
N1 Anxiety	0.180	**0.596**	0.062	−0.139	−0.049
O7 Variety	0.113	0.065	**0.717**	0.050	−0.030
O6 Reflection/intellect	0.220	−0.061	**0.695**	0.026	−0.028
O4 Ingenuity	−0.099	−0.009	**0.645**	−0.024	0.099
Co6 Order	0.061	0.021	−0.035	**0.751**	−0.035
Co1 Reliability/responsibility	0.008	−0.025	0.005	**0.717**	0.002
Co4 Efficiency/productiveness	−0.052	−0.212	0.099	**0.484**	0.194
Ag3 Understanding/compassion	0.027	−0.129	0.019	−0.097	**0.682**
Ag4 Gentleness	−0.022	0.109	−0.006	0.126	**0.663**
Ag1 Trust	0.189	0.002	0.034	0.073	**0.601**

*Bold values refer to item loadings on targeted factors.*

We then did a CFA of the 15 items in Table 3. We used the WLSMV estimation method, since all items were three-category ordinal variables. The values of the key goodness-of-fit indices for the respective simple structure five-factor model were as follows: robust χ^2^ = 166.401 (df = 80, *p* = 0.000), robust CFI = 0.946, robust TLI = 0.930, robust RMSEA = 0.027 (90% CI = [0.021–0.033], p[RMSEA < 0.05] = 1.000), and SRMR = 0.042. These results are slightly better than those reported for the BFI-2 administered to Turkish students by Cemalcilar et al. (2021). Those authors report that their community sample yielded even lower psychometric quality. Compared to that non-WEIRD performance, our results are quite satisfactory.

**FIGURE 1:**
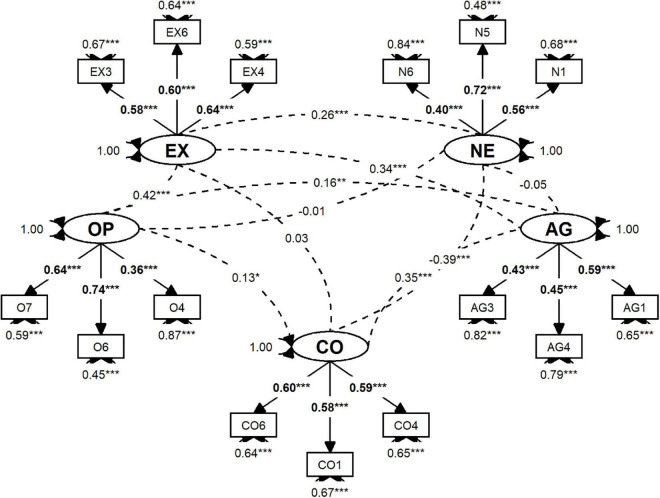
The five-factor CFA model of personality based on the sample of 1500 adult Mongolians.

Figure 1 shows the parameter estimates for our CFA model. We note that some items have fairly low loadings, suggesting that our Big Five tool may need further optimization in the Mongolian context, such as a search for a few better-performing items. Yet, the model possesses a relatively high level of discriminant validity so the revealed factors can indeed be considered distinct dimensions of personality among Mongolians. For more details please see Appendix 1 in the online Supplementary material.

None of the CFA analyses across members of groups where the Big Five may be expected to emerge more clearly (university-educated, or urban, or relatively wealthy, or online respondents) produced a Big Five model with overall superior psychometric properties compared to the results from the general population (whole sample).

The model for online respondents had a slightly better fit (marginally significant χ^2^ statistic, higher values of CFI and TLI, and a lower value of RMSEA) but was more problematic in terms of factor loading sizes (less balanced loadings; more items with loadings lower than 0.40; poorer reliability estimates) than the full-sample model. Other sub-group models were inferior to the full-sample model both in terms of model fit and construct validity. Details are provided in the Supplementary material.

Importantly, we also found that the proposed model performs more or less equivalently across different socio-demographic groups in Mongolia. The same factor structures emerge among respondents with and without higher education, rural and urban dwellers, respondents with low/medium or high income, online and F2F respondents, and female and male respondents. Measurement model parameters were also reasonably equivalent across those groups, especially factor loadings. Full invariance of item thresholds was supported to a lesser extent, but even in that respect, our results did not suggest dramatic differences across groups for any grouping variables (see Appendix 3 in Supplementary material for more details on measurement invariance analysis).

Next, we factor-analyzed (PCA) the 15 items with promax rotation (kappa 2) and then factor-analyzed the oblique factors. We obtained two components with eigenvalues over 1.00, explaining 24.9 and 23.5% of variance. The solution is provided in Table 4. It clearly demonstrates a plasticity component and a stability component (DeYoung, 2006). A hierarchical CFA model, reported in Appendix 4 in the Supplementary material, also suggested that the second-order stability and plasticity factors are recognizable among Mongolians, although not as clearly as in WEIRD countries.

**TABLE 4:** Solution of the exploratory principal components analysis of the five oblique Big Five factors.

	Component	
	1 Plasticity	2 Stability
E	**0.795**	−0.191
O	**0.693**	0.213
C	0.031	**0.671**
N	0.200	−**0.597**
A	0.243	**0.564**

*Bold values refer to item loadings on targeted factors.*

Age was positively correlated with C (*r* = 0.21), and A (*r* = 0.16), and negatively with O (*r* = -0.26), E (*r* = -0.12), and N (*n* = -0.08), all correlations significant at *p* < 0.01. Women (*n* = 770) scored higher on neuroticism than men (*n* = 730), *p* = 0.001. No significant gender differences emerged on any other factor.

## Discussion

A Big Five tool adapted to Mongolian culture worked reasonably well across our nationally representative sample of Mongolia. It also yielded a more or less clear Big Two. These results suggest that when Mongolian personality is assessed with a locally adapted etic tool, its structure is similar to that of WEIRD nations. Associations of the Big Five with culture and gender also suggest that the Mongolian patterns are not markedly different from those observed in WEIRD nations. However, unlike previous studies in non-WEIRD nations, we did not find that more educated, wealthier, urban, or online Respondents yield a clearer Big Five model than the whole, nationally representative sample. The same factor structure, with more or less the same measurement parameter values, emerge in various socio-demographic groups reflected in our sample. This is a curious exception to what has been reported so far from other studies in non-WEIRD nations, suggesting that generalizations across all non-WEIRD respondents from all cultures may be inappropriate.

It may be that our adaptation of our Big Five tool to the Mongolian context has made it more meaningful to all Mongolians than it would have been if fielded in a prefabricated version. This may suggest a specific solution to the problem of low-quality results when WEIRD etic psychometric tools are used in non-WEIRD nations. One way to achieve optimal results is to focus on demographic groups that are more comfortable with such tools. However, that approach excludes large sections of the population. Our study suggests a different approach: local adaptation of the research tool through focusing on items that make sense in the local cultural environment and do not compromise the psychometrical properties of the tool to an unacceptable degree. Further research is needed to ascertain if our approach would work outside Mongolia.

One limitation of our study is that it cannot answer an interesting question. Is our tool (or any other Big Five tool) better for the Mongolian context than an alternative personality tool, such as HEXACO? Next, would an emic tool (starting from a local personality lexicon) be a better tool, first in terms of internal structure, and, second, in terms of external linkages? In future research, it would be interesting to compare the predictive properties of Big-Five or HEXACO tools and emic personality tools in non-WEIRD countries, such as Mongolia.

## Data Availability Statement

The raw data supporting the conclusions of this article will be made available by the authors, without undue reservation.

## Ethics statement

Ethical review and approval were not required for the study on human participants in accordance with the local legislation and institutional requirements. The patients/participants provided their written informed consent to participate in this study.

## Author contributions

MM: study design, data analysis, and manuscript writing. BS: data analysis and manuscript writing. MT, EJ, and MS: data collection. AK: manuscript writing. All authors contributed to the article and approved the submitted version.

## Conflict of Interest

EJ was employed by SICA. The remaining authors declare that the research was conducted in the absence of any commercial or financial relationships that could be construed as a potential conflict of interest.

## Publisher’s Note

All claims expressed in this article are solely those of the authors and do not necessarily represent those of their affiliated organizations, or those of the publisher, the editors and the reviewers. Any product that may be evaluated in this article, or claim that may be made by its manufacturer, is not guaranteed or endorsed by the publisher.
