# Erratum: The big five and big two personality factors in Mongolia

**DOI:** 10.3389/fpsyg.2022.1042351

**Published:** 2022-09-28

**Authors:** Frontiers Production Office

**Affiliations:** Frontiers Media SA, Lausanne, Switzerland

**Keywords:** Big Five, Big Two, Mongolia, culture, personality

Due to a production error, there was a mistake in the author list order and affiliations two and three as published. The correct author list and affiliation list are as follows:

Michael Minkov^1, 2, 3*^, Boris Sokolov^3^, Marc Albert Tasse^4^, Erdenebileg Jamballuu^4^, Michael Schachner^5^ and Anneli Kaasa^2^

^1^ Varna University of Management, Sofia, Bulgaria

^2^ University of Tartu, Tartu, Estonia

^3^ Ronald Inglehart Laboratory for Comparative Social Research, National Research University Higher School of Economics, Moscow, Russia

^4^ SICA, Ulaanbaatar, Mongolia

^5^ Vrije Universiteit, Amsterdam, Netherlands

The publisher apologizes for this mistake. The original version of this article has been updated.

