# Supplementary material for: The Big Five and Big Two personality factors in Mongolia
Source: Front Psychol. 2022 Jul 26;13:917505. doi: 10.3389/fpsyg.2022.917505 (PMC9361345; doi:10.3389/fpsyg.2022.917505)
Supplement: Supplementary file 1 [file Data_Sheet_1.docx]

**Supplementary Materials to “The Big Five in Mongolia”**

***Appendix 1. Confirmatory factor analysis of 15 personality items***

For this analysis, we used three items for each of the five standard personality factors. Items were selected based on the results of EFA of the full set of items, using the EFA factor loadings as a selection criterion. For the CFA, we assumed a simple model structure (no cross-loadings and no residual covariances between items). Since all 15 personality items are three-category ordinal variables, we used the weighted least squares mean and variance adjusted (WLSMV) estimator, the default option for categorical CFA in many software packages, as implemented in the R package lavaan (v.0.6-9; Rosseel 2012). Consequently, the robust versions of the χ^2^ statistics and the key global fit indices (CFI, TLI, and RMSEA) were used to assess model fit (Brosseau-Liard *et al.,* 2012; Brosseau-Liard and Savalei, 2014; Savalei, 2018). No missing values on the 15 items were present in the data.

The robust χ^2^ statistics for the proposed model was equal to 166.401, with df = 80 (p < 0.001). The significant χ^2^ statistic may suggest poor model fit, but the sample size is quite large (N = 1,500), and it is well known that the χ^2^ statistics is oversensitive to even minor model misspecifications in large samples. Other global fit indices suggest that the five-factor model fits the Mongolian data reasonably well, though not perfectly. The robust Comparative Fit Index (CFI) and the robust Tucker Lewis Index (TLI) were equal to 0.946 and 0.930, respectively. It is typically considered that values of these two indices higher than 0.900 or 0.950 indicate acceptable model fit (Brown 2015, pp. 73-75; Hu and Bentler, 1999; Kline, 2015, p. 209). The robust Root Mean Square Error of Approximation (RMSEA) was equal to 0.027, with a 90% confidence interval of 0.021 – 0.033, whereas popular SEM textbooks state that the RMSEA values lower than 0.05 indicate a good model fit (Browne & Cudeck 1993; Kline 2015, 206; Brown, 2015, p. 73-75). Additionally, the probability that the population RMSEA value is lower than 0.05 (the so called close-fit hypothesis) was equal to 1.000. Finally, the Standardized Root Mean Square Residual (SRMR) was equal to 0.042, with values lower than 0.08 indicating good fit (Hu & Bentler, 1999; Brown, 2015; Asparouhov & Muthén, 2018). So, in terms of global fit, the proposed five-factor model can be considered acceptable, since no one global fit index, with the single exception of the χ^2^ statistics, had unsatisfactory values for this model.

The model is nevertheless not free from some local misspecifications. The parameters with the largest standardized Expected Parameter Change (EPC) values were the residual covariance between N5 and N1 (std. EPC = –0.377), CO6 and CO1 (std. EPC = 0.311), CO1 and CO4 (std. EPC = –0.219), and N6 and N5 (std. EPC = 0.203). This indicates that some non-negligible portion of covariance between several Neuroticism and Conscientiousness items might be caused by something else than the respective constructs. There were also some residual covariances between items belonging to different theoretical dimensions that exceeded 0.10, a value which is sometimes recommended as a threshold for substantively misspecified residual covariances (Saris, Satorra, & van der Veld, 2009, p. 571). The largest one was the covariance between E3X and N1 (std. EPC = 0.15). On the other hand, no cross-loading had a value larger than the threshold of 0.40 proposed by Saris et al. (2009). Additionally, no cross-loading was identified as a significant misspecification by the advanced misspecification detection method developed by Saris et al. (2009)

In terms of construct validity, only two factors had all three indicators exhibiting relatively high, and at the same time balanced, standardized loadings (std. λs): Extraversion (std. λs = 0.58 – 0.64) and Conscientiousness (std. λs = 0.58 – 0.60). One indicator of Openness (O4; std. λ = 0.36) and one indicator of Neuroticism (N6; std. λs = 0.40) had their respective standardized loadings no higher than 0.40, and much smaller than the loadings of other items related to the respective constructs (see Figure 1 in the main text). The Cronbach’s alphas (none > 0.51), as well as their ordinal counterparts (Zumbo et al. 2007; none > 0.64), various versions of the omega coefficient (none > 0.58) and the average variance extracted (AVE; none > 0.37), were low, both for particular factors and for the full model. Nevertheless, this is not surprising, in view of the small number of items per factor and the obvious violation of key assumptions of the aforementioned reliability measures (e.g. tau-equivalence in case of the alpha coefficient). The item-specific R^2^s varied from 0.13 (O4) to 0.55 (O6), but all but two were smaller than 0.5.

Although the model does not perform greatly in terms of its overall construct validity, it still possesses a quite reasonable level of discriminant validity. All pairwise inter-factor correlations were lower than 0.50 in absolute values, the largest one being between Extraversion and Openness: 0.42. At the same time, the square root of the smallest individual-factor average variance extracted coefficient was 0.496 (that of Agreeableness, whose AVE was equal to 0.246), larger than any pairwise inter-factor correlation in the model, thus satisfying the so-called Fornell–Larcker criterion (Fornell & Larcker, 1981). Finally, two discriminant validity tests recommended by Rönkkö and Cho (2020) - CI_CFA_ (cut) and χ^2^ (cut) - also suggested that the model distinguishes between all five factors quite well. This resonates well with the previously reported finding of no significantly misspecified cross-loadings, and suggests that the standard Big Five personality dimensions can indeed be distinguished in the Mongolian sample using the proposed instrument.

Importantly, our goal in the current study was not to produce a fully reliable measurement personality inventory for the Mongolian population, but instead to show that the standard Big Five personality dimensions can be distinguished in a population that has not been previously studied by personality researchers. The CFA model reported here seems to fit this purpose quite well.

***Appendix 2. Confirmatory factor models for various socio-demographic groups***

As it is mentioned in the main text, some previous studies revealed that one may expect to find more consistent, robust, and reliable personality factor structures in certain types of populations (in cross-national analyses) or population groups (in within-country analyses), usually in richer or more educated ones. In our Mongolian data set, we identified four demographic groups which might yield clearer factor solutions than the full sample: (a) respondents with higher education (possessing a Bachelor degree or above), (b) urban dwellers (non-herders), (c) online respondents, and (d) high-income respondents (those with monthly income is more than a million tugriks, i.e. about 300 EUR, which is roughly the income of the richest one third of the sample.). We estimated the five-factor CFA model, described in Appendix 1, separately for each of those four subsamples.

Contrary to our expectations, we found that no sub-sample model simultaneously had both better fit measure values and stronger internal consistency than the full-sample model. The model for online respondents had a slightly better fit (marginally significant χ^2^ statistic, higher values of CFI and TLI and a lower value of RMSEA), but was certainly more problematic in terms of factor loading sizes (less balanced loadings; more items with loadings lower than 0.40; poorer reliability estimates) than the full-sample model. Other sub-group models were inferior to the full-sample model both in terms of model fit and construct validity (see Table A2.1 and Figures A2.1-4).

Table A2.1. CFA of the Five-Factor Personality Model in Four Demographic Groups: Model Fit

| Group (N) | Robust χ^2^  (df, p-value) | Robust CFI | Robust TLI | Robust RMSEA [90% CI]  p < 0.05 | SRMR |
| --- | --- | --- | --- | --- | --- |
| Higher education (483) | 125.363  (80, 0.001) | 0.909 | 0.880 | 0.034  [0.022 – 0.045]  0.991 | 0.062 |
| Urban dwellers (1128) | 172.851  (80, 0.000) | 0.929 | 0.907 | 0.032  [0.026 – 0.039]  1.000 | 0.049 |
| Online  (738) | 100.987  (80, 0.057) | 0.965 | 0.954 | 0.019  [0.000 – 0.029]  1.000 | 0.050 |
| High income  (547) | 113.526  (80, 0.008) | 0.940 | 0.921 | 0.028  [0.015 – 0.039] 1.000 | 0.055 |

Figure A2.1 CFA parameter estimates: Respondents with higher education


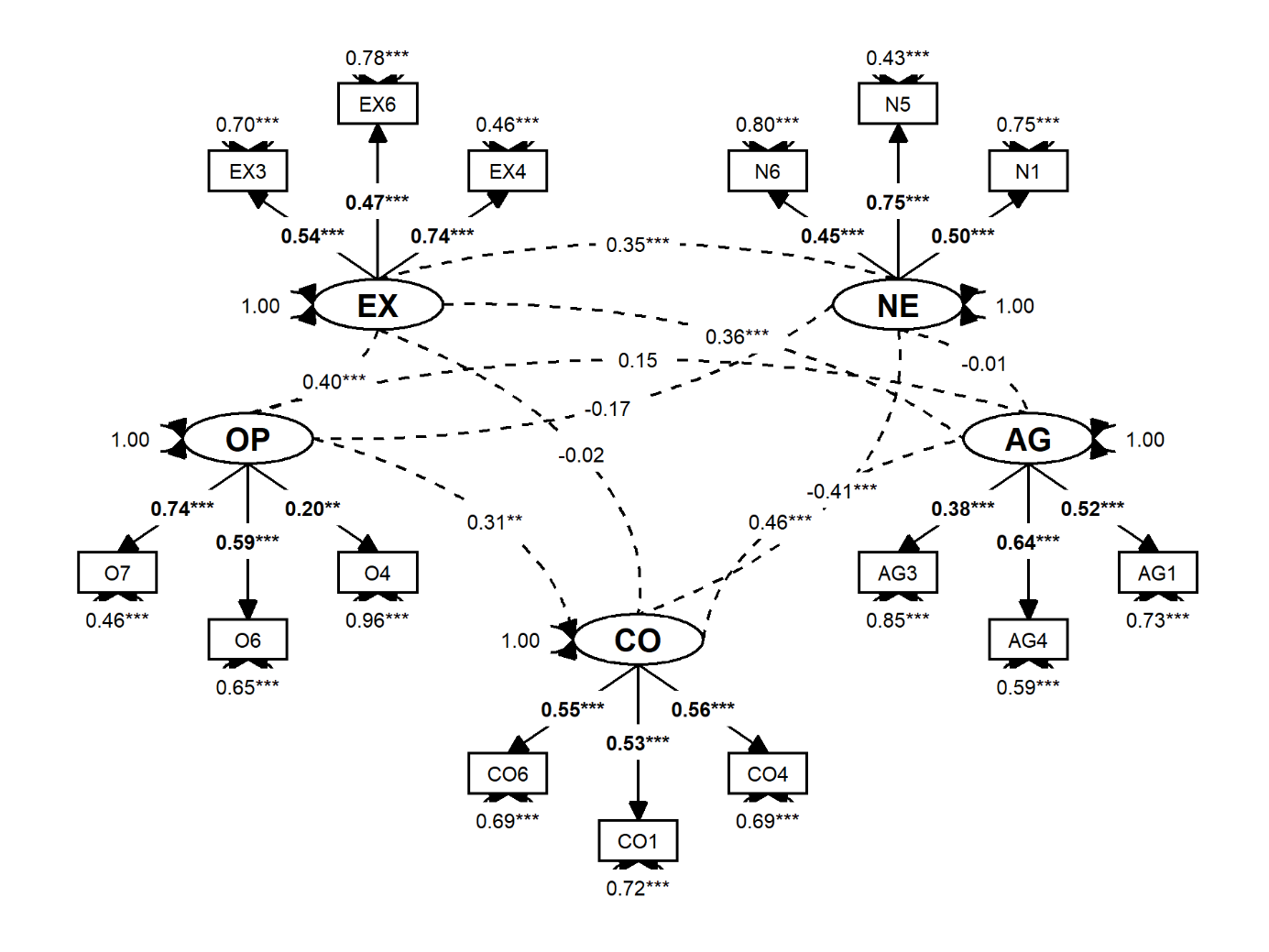


*Note*: Ovals represent latent factors, rectangles respresent observed indicators. Bold numbers on solid single-headed arrows going from factors to indicators show the estimated values of respective factor loadings. Numbers on double-headed arrows around indicators and factors show the estimated values of indicator residual variances and latent variances respectively. Finally, numbers on dashed double-headed arrows between factors show the estimated values of latent correlations. All shown parameters are standardized.

*** p-value < 0.001, ** p < 0.01, * p < 0.05.

Figure A2.2 CFA parameter estimates: Urban dwellers (non-herders)
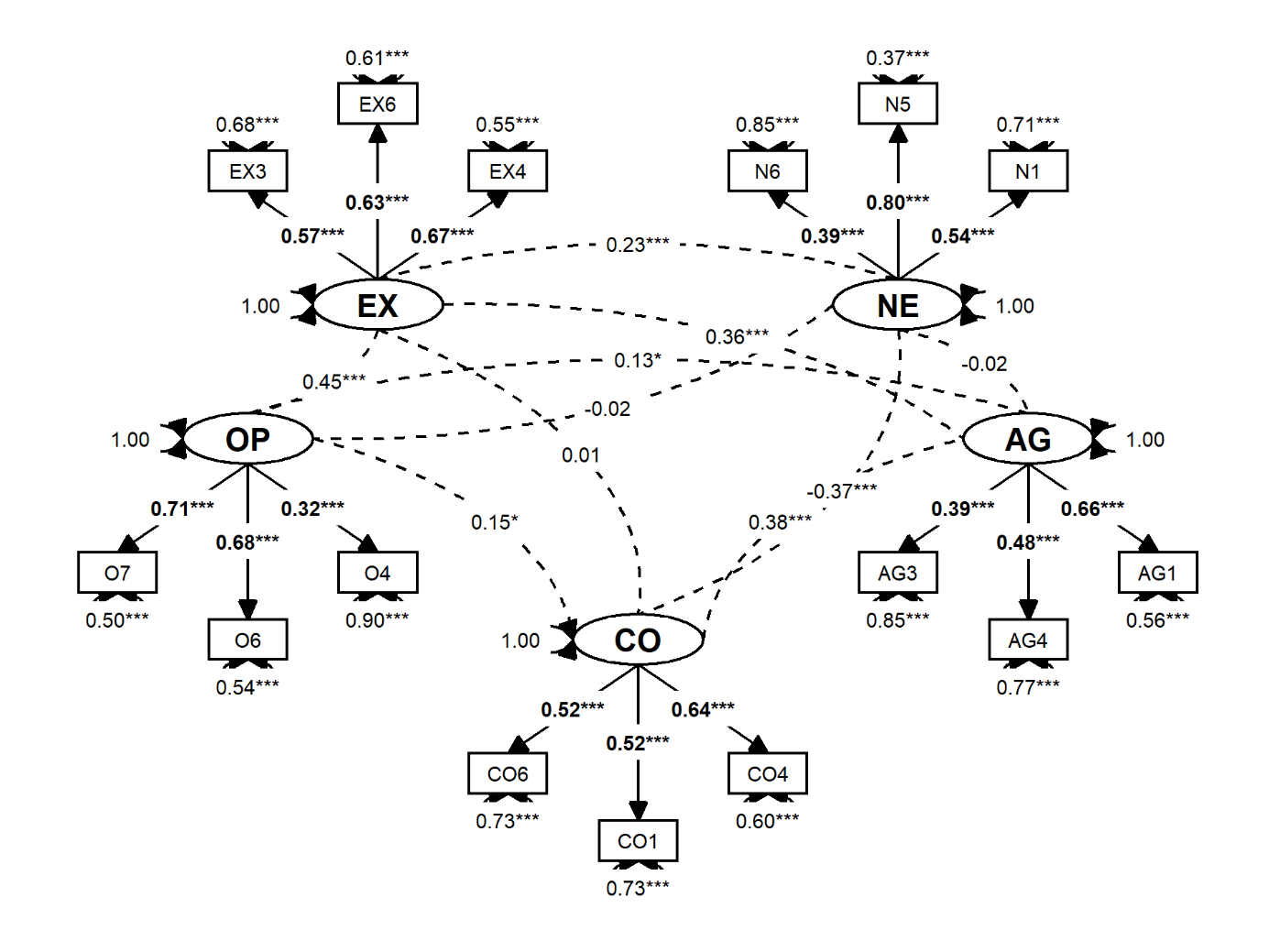


Figure A2.3 CFA parameter estimates: High-income respondents


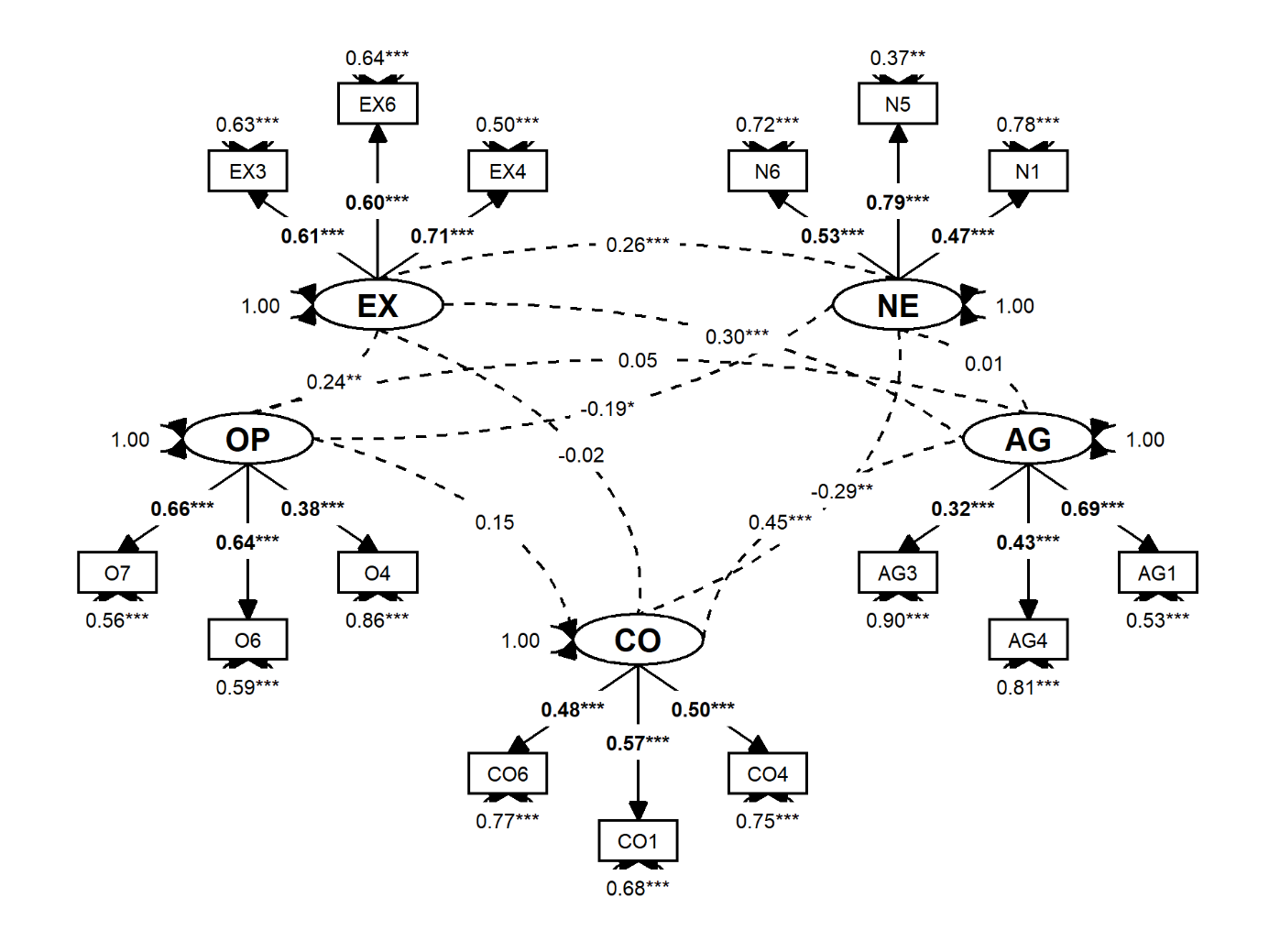


Figure A2.4 CFA parameter estimates: Online respondents


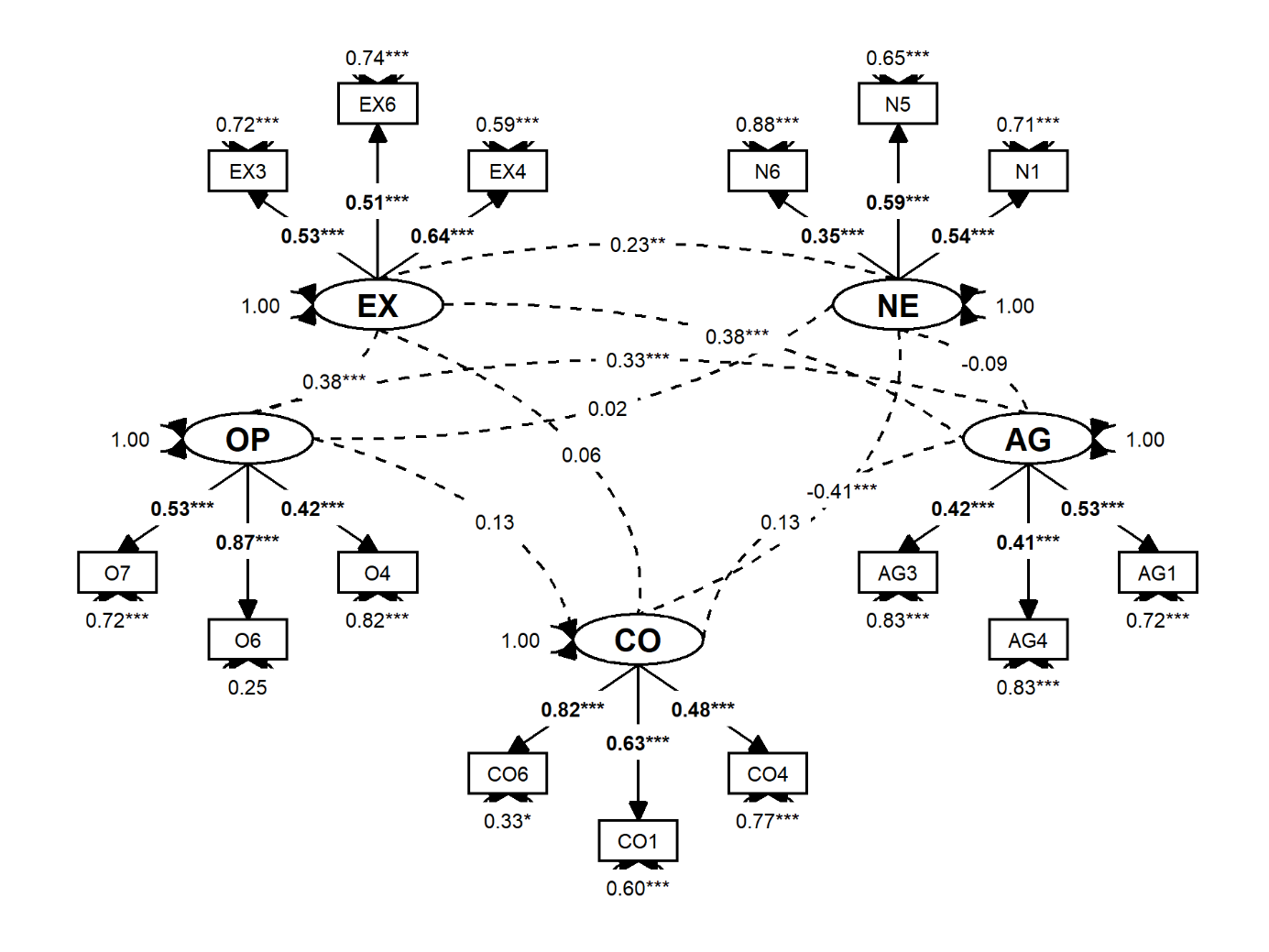


***Appendix 3. Measurement invariance analysis of the 5-factor personality model across different socio-demographic groups.***

We complemented the sub-group analyses reported in Appendix 2 by a set of measurement invariance (MI) tests for five different grouping structures defined using the following socio-demographic variables: (a) respondents without higher education vs. respondents with higher education (operationalization details for grouping variables a-d can be found in the previous section), (b) rural vs. urban dwellers (herders vs. non-herders), (c) online respondents vs. face-to-face respondents, and (d) low/medium respondents vs. high income respondents, as well as (e) male vs. female respondent. We added the latter grouping variable because we compare means on personality factors across female and male respondents in the main text and below in Appendix 5, so it is important to ensure that the measurement model returns numerically equivalent estimates of mean scores for these two subsamples.

To save space and time, we do not provide a detailed account of MI and assume that interested readers are already familiar, at least briefly, with key MI concepts, terminology, and existing standards for MI testing in applied studies. Informative introductions to the MI testing literature may be found elsewhere (e.g. Davidov et al. 2014; Putnik & Bornstein 2016).

To test the proposed Big Five personality model for MI across different socio-demographic groups, we utilized the multiple-group confirmatory factor analysis (MGCFA) framework. Since all our BF items are three-point categorical scales, we used the WLSMV estimator and followed the MI testing method recommended by Wu and Estabrook (2016). Those authors suggest that, contrary to the common practice, invariance of item thresholds should be tested before invariance of loadings. Moreover, with binary and three-category indicators the former type of invariance (of thresholds), is not testable, so the only assumption we did actually test for our Big Five model was that item loadings are sufficiently similar across various socio-demographic groups in Mongolia. For all five grouping variables, the results were quite good: the χ^2^ difference test (which is usually almost always significant in large samples) returned p-values larger than 0.1 for every grouping structure and the differences in the values of alternative global fit indices (namely, CFI, RMSEA, and SRMR) between the loadings-and-thresholds-invariant model and the thresholds-only-invariant model were all extremely small^[[1]](#footnote-1)^ (see tables A3.1 – 3.5 below).

We then complemented that analysis by conducting more traditional categorical MI tests, where one sequentially estimates three models, assuming different levels of invariance: configural invariance (equivalent factor structures in different groups), weak invariance (equivalent factor loadings), and strong invariance (equivalent loadings and equivalent item thresholds). The traditional approach yielded a slightly less excellent results than the Wu-Estabrook approach, although we believe that it does not suggest dramatic differences in factor structures across the various subsamples of our Mongolian data set. For most grouping variables, invariance of factor loadings (weak invariance) is still a reasonable assumption, supported by all global fit indices, except for the χ^2^ difference test. However, the CFI does not support metric invariance for herders vs. non-herders (ΔCFI = – 0.013, compared to the configural model, which is somewhat larger than the 0.1 cut-off proposed in Chen, 2007). Yet, the RMSEA and the SRMR values both suggest the opposite conclusion, as well as visual assessment of the differences in the loading sizes across various groups^[[2]](#footnote-2)^.

As to strong invariance, according to the CFI and the χ^2^ test, it may be a problematic assumption for herders vs. non-herders, respondents with vs/ without higher education, and online vs. F2F respondents. Nevertheless, the RMSEA and the SRMR differences did not exceed 0.003 for any grouping variable, which suggests that differences in threshold sizes across various groups are not very large. Anyway, it seems that one could obtain less critical CFI differences between the metric and the scalar model for problematic groupings with so-called partial invariance tests (when a few thresholds are allowed to be non-equivalent across groups), but since the MI analysis is certainly not the main goal of our study, we preferred to stop at this point. All in all, the Wu-Estabrook approach reveals no significant global differences in loading sizes between any grouping structures of interest, and the traditional approach indicates relatively minor differences, and mostly for threshold parameters. Thus, it seems legitimate to conclude that the proposed Big Five model of personality performs in a more or less similar way in different subgroups of the Mongolian population.

Table A3.1. Measurement invariance across respondents with vs. without higher education. Fit statistics for model assuming different levels of invariance.

| **Wu-Estabrook approach** | | | | | | |
| --- | --- | --- | --- | --- | --- | --- |
| *Model* | *scaled χ^2^* | *df* | *p-value* | *Scaled*  *CFI* | *Scaled RMSEA* | *Scaled SRMR* |
| Configural + Equal thresholds | 249.706 | 160 | 0.000 | 0.942 | 0.027 | 0.051 |
|  | *Δ χ^2^ (robust)* | *Δ df* | *p-value* | *Δ Scaled*  *CFI* | *Δ Scaled RMSEA* | *Δ Scaled SRMR* |
| \|+ Equal loadings | 10.609 | 10 | 0.389 | – 0.001 | 0..000 | 0.000 |
| **Traditional approach** | | | | | | |
| Configural | 249.706 | 160 | 0.000 | 0.942 | 0.027 | 0.051 |
| \|+ Equal loadings | 19.103 | 10 | 0.019 | – 0.008 | 0.001 | 0.002 |
| + Equal thresholds | 39.894 | 10 | 0.000 | – 0.018 | 0.003 | – 0.001 |

*Note*: Respondents with higher education are defined as those possessing a Bachelor degree or above. Tables A3.1-5 show the absolute values of fit indices for the configural model. For models assuming more demanding levels of invariance, we show the differences in absolute values of the respective indices compared to models with less demanding invariance levels. For all fit indices, both the absolute values and the differences between model assuming different levels of invariance are based on the scaled version of the χ^2^. The only exception is the Δ χ^2^, which is based on the correction proposed by Satorra (2000), and is the function of the two standard χ^2^ statistics. All computations were performed in the R packages *lavaan* (v. 0.6-9.0) and *semTools* (v. 0.5-5).

Table A3.2. Measurement invariance across rural vs. urban dwellers (herders vs. non-herders). Fit statistics for model assuming different levels of invariance.

| **Wu-Estabrook approach** | | | | | | |
| --- | --- | --- | --- | --- | --- | --- |
| *Model* | *scaled χ^2^* | *df* | *p-value* | *Scaled*  *CFI* | *Scaled RMSEA* | *Scaled SRMR* |
| Configural + Equal thresholds | 257.096 | 160 | 0.000 | 0.938 | 0.028 | 0.052 |
|  | *Δ χ^2^ (robust)* | *Δ df* | *p-value* | *Δ Scaled*  *CFI* | *Δ Scaled RMSEA* | *Δ Scaled SRMR* |
| \|+ Equal loadings | 14.833 | 10 | 0.138 | – 0.004 | 0..000 | 0.000 |
| **Traditional approach** | | | | | | |
| Configural | 257.096 | 160 | 0.000 | 0.938 | 0.028 | 0.052 |
| \|+ Equal loadings | 21.239 | 10 | 0.039 | – 0.013 | 0.002 | 0.003 |
| + Equal thresholds | 29.701 | 10 | 0.000 | – 0.015 | 0.002 | – 0.001 |

Table A3.3. Measurement invariance across online vs. F2F respondents. Fit statistics for model assuming different levels of invariance.

| **Wu-Estabrook approach** | | | | | | |
| --- | --- | --- | --- | --- | --- | --- |
| *Model* | *scaled χ^2^* | *df* | *p-value* | *Scaled*  *CFI* | *Scaled RMSEA* | *Scaled SRMR* |
| Configural + Equal thresholds | 298.207 | 160 | 0.000 | 0.917 | 0.034 | 0.056 |
|  | *Δ χ^2^ (robust)* | *df* | *p-value* | *Δ Scaled*  *CFI* | *Δ Scaled RMSEA* | *Δ Scaled SRMR* |
| \|+ Equal loadings | 11.138 | 10 | 0.347 | – 0.002 | – 0.001 | 0.000 |
| **Traditional approach** | | | | | | |
| Configural | 298.207 | 160 | 0.000 | 0.917 | 0.034 | 0.056 |
| \|+ Equal loadings | 20.243 | 10 | 0.027 | – 0.008 | 0.000 | 0.003 |
| + Equal thresholds | 60.635 | 10 | 0.000 | – 0.026 | 0.003 | – 0.001 |

Table A3.4. Measurement invariance across low/ medium vs. high-income respondents. Fit statistics for model assuming different levels of invariance.

| **Wu-Estabrook approach** | | | | | | |
| --- | --- | --- | --- | --- | --- | --- |
| *Model* | *scaled χ^2^* | *df* | *p-value* | *Scaled*  *CFI* | *Scaled RMSEA* | *Scaled SRMR* |
| Configural + Equal thresholds | 248.767 | 160 | 0.000 | 0.944 | 0.027 | 0.052 |
|  | *Δ χ^2^ (robust)* | *df* | *p-value* | *Δ Scaled*  *CFI* | *Δ Scaled RMSEA* | *Δ Scaled SRMR* |
| \|+ Equal loadings | 11.931 | 10 | 0.290 | – 0.002 | 0.000 | 0.000 |
| **Traditional approach** | | | | | | |
| Configural | 248.767 | 160 | 0.000 | 0.944 | 0.027 | 0.052 |
| \|+ Equal loadings | 15.763 | 10 | 0.107 | – 0.005 | 0.000 | 0.002 |
| + Equal thresholds | 18.955 | 10 | 0.041 | – 0.006 | 0.001 | – 0.001 |

*Note*: Respondents with higher education are defined as those with monthly income is more than a million tugriks, i.e. about 300 EUR (which is roughly the income of the richest one third of the sample.)

Table A3.5. Measurement invariance across female vs. male respondents. Fit statistics for model assuming different levels of invariance.

| **Wu-Estabrook approach** | | | | | | |
| --- | --- | --- | --- | --- | --- | --- |
| *Model* | *scaled χ^2^* | *df* | *p-value* | *Scaled*  *CFI* | *Scaled RMSEA* | *Scaled SRMR* |
| Configural + Equal thresholds | 273.111 | 160 | 0.000 | 0.929 | 0.031 | 0.054 |
|  | *Δ χ^2^ (robust)* | *df* | *p-value* | *Δ Scaled*  *CFI* | *Δ Scaled RMSEA* | *Δ Scaled SRMR* |
| \|+ Equal loadings | 8.861 | 10 | 0.545 | – 0.001 | – 0.001 | 0.000 |
| **Traditional approach** | | | | | | |
| Configural | 273.111 | 160 | 0.000 | 0.929 | 0.031 | 0.054 |
| \|+ Equal loadings | 11.154 | 10 | 0.107 | – 0.001 | – 0.001 | 0.001 |
| + Equal thresholds | 16.356 | 10 | 0.041 | – 0.005 | 0.000 | 0.000 |

***Appendix 4. Hierarchical factor analysis of 15 personality items.***

EFA of the five factors from the 15 personality items using oblique rotation, reported in the main text, clearly suggests that the five personality dimensions can be further decomposed into two larger dimensions, stability and plasticity, sometimes referred to as the Big Two. We checked that exploratory finding by estimating a hierarchical CFA model with the Big Five as first-order factors and the Big Two as second-order factors. However, the baseline specification, where Neuroticism, Conscientiousness, and Agreeableness belong to the Stability factor, and Extraversion and Openness belong to the Plasticity factor, did not fit the data well. The key goodness-of-fit statistics for that model had the following values: robust χ^2^ = 296.813 (df = 84, p = 0.000), robust CFI = 0.868, robust TLI = 0.835, robust RMSEA = 0.041 (90% CI = [0.036 - 0.046], p[RMSEA < 0.05] = 0.998), SRMR = 0.061. The CFI and TLI values were considerably lower than even 0.90, which is often recommended as a liberal threshold of acceptable model quality (see references above, in Appendix 1). In addition, the estimated residual variance of Extraversion and the estimated factor variance of Plasticity were negative, which is implausible since any variance parameter by definition may take only non-negative values. These observations indicate that the proposed hierarchical CFA model does not capture the covariance patterns for the first-order factors properly.

Figure A4.1. Pairwise inter-factor correlations from a 5-factor CFA model of 15 personality items.


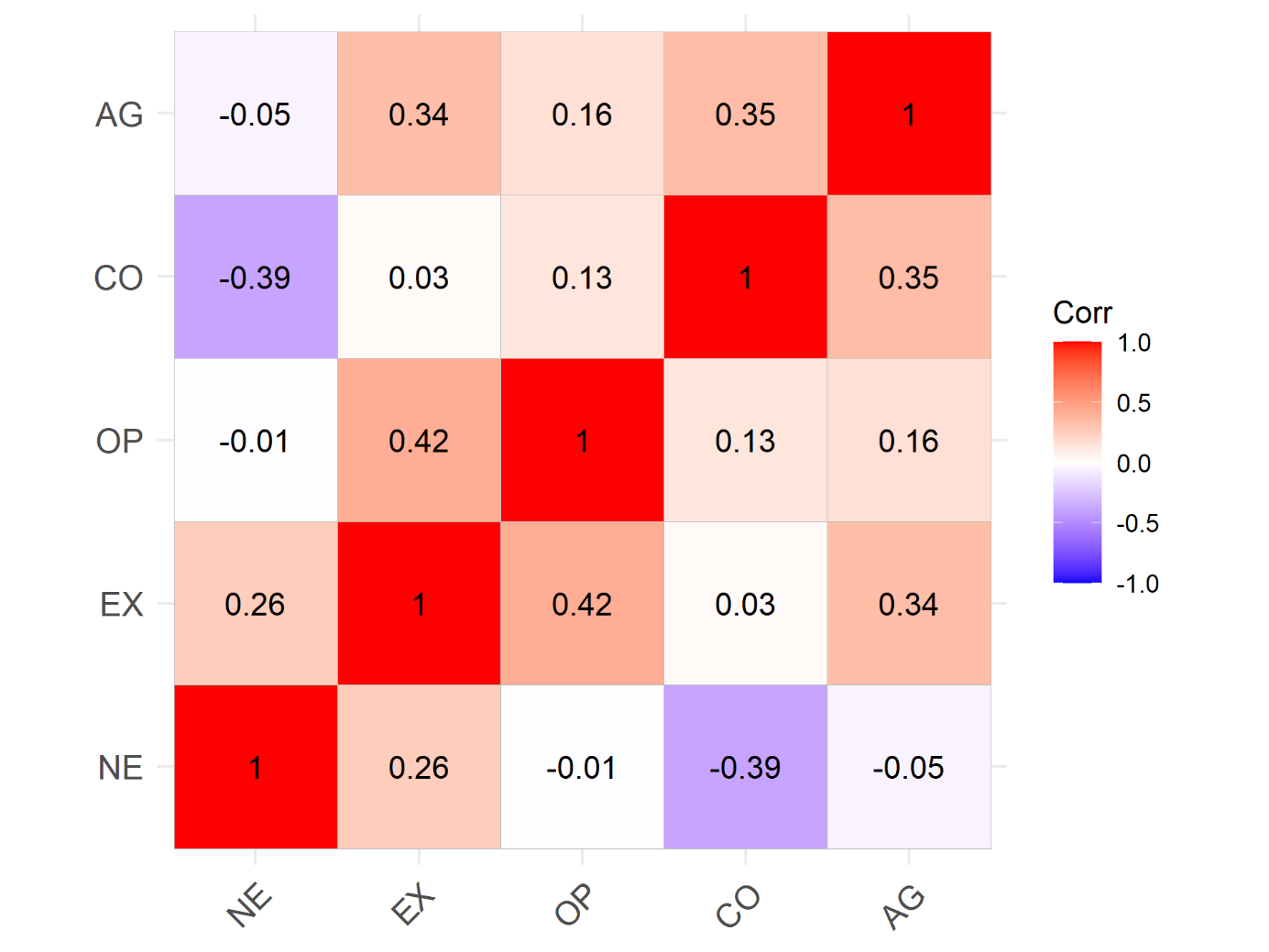


The problems with the two-factor hierarchical model become clear from Figure A4.1, which shows pairwise inter-factor correlation estimates from the CFA model described in the main text and in Appendix 1. There are two pairs of correlated first-order factors, Agreeableness + Conscientiousness, and Extraversion + Openness, but Neuroticism obviously stands alone: it correlates negatively with Conscientiousness, but is uncorrelated with Agreeableness. Also, it is unrelated to Openness but correlates positively with Extraversion. After trying several alternative specifications in a semi-exploratory fashion, we managed to find one with somewhat better fit and reasonable loading sizes for the second-order factors. That model included the same two second-order factors, but with only two related first-order factors each. The first-order Neuroticism factor did not belong to any of the second-order factors but was instead allowed to correlate with both. That model also implied an additional equality restriction on the factor loadings of Extraversion and Openness on the Plasticity factor.

That model had the following values of the fit indices: robust χ^2^ = 254.075 (df = 84, p = 0.000), robust CFI = 0.894, robust TLI = 0.868, robust RMSEA = 0.037 (90% CI = [0.032 – 0.042], p[RMSEA < 0.05] = 1.000), SRMR = 0.053. The CFI and TLI values were still relatively poor, but better than those for the baseline model. That model also had relatively high second-order loadings: the standardized loadings for Extraversion and Openness were both equal to 0.648, while for Agreeableness and Conscientiousness they were equal to 0.563 and 0.609 respectively. Neuroticism correlated with the reduced Stability factor at –0.386 and with the Plasticity factor at 0.202. The correlation between the second-order factors was 0.440.

Although not providing as clear a picture as the exploratory analysis of the Mongolian data, the hierarchical CFA results still reveal that there are indeed two higher-order personality factors, which resembles the Big Two model quite closely, with one exception: Neuroticism does not belong to any second-order factor. Yet, its association with the theoretical second-order factor, Stability, is quite strong and in the expected direction (i.e. negative). Since Mongolia is a non-WEIRD country, it is hard to expect much better results. Taken together, our EFA and CFA results do suggest that the two-factor higher-order model of personality seems to hold in the Mongolian population. The fact that there are some deviations from the WEIRD-based standard model is not especially critical, in our view.

***Appendix 5: Questionnaire***

Note: All items have an in-between response option ("I am somewhere in-between these two") shown to the respondents but not shown in the questionnaire below.

**Neuroticism**

| N1. I worry a lot and often feel nervous. | I am usually relaxed and do not worry much. |
| --- | --- |
| N2. I easily get angry. | It is hard to make me angry. |
| N3. During most of my life as an adult person, I have often felt tired even without working very hard. | During most of my life as an adult person, I have been able to work hard without feeling tired. |
| N4. I often feel sad. | I rarely feel sad. |
| N5. I get rattled in difficult situations. | I stay calm in difficult situations. |
| N6. I am often afraid that I will do something wrong or stupid and people will laugh at me. | I am rarely afraid that I will do something wrong or stupid. |

**Extraversion**

| Ex1. I like to meet new people and talk to them. | I rarely have an interest in meeting new people and talking with them. |
| --- | --- |
| Ex2. I am usually bold. | I am usually shy. |
| Ex3. I often talk a lot. | I usually keep quiet. |
| Ex4. I like celebrations and parties with a lot of music and laughing. | I prefer to have peace and quiet. |
| Ex5. I am a lively person, full of energy. | I often seem to be slow or sleepy, without much energy. |
| Ex6. I often laugh. | I rarely laugh. |

**Conscientiousness**

| Co1. I arrive exactly on time for meetings or other events. I am hardly ever late. | I am often late and people have to wait for me. |
| --- | --- |
| Co2. I always think a lot before an important decision: getting married, having children, changing my job, etc. | I often make important decisions and act without thinking too much. |
| Co3. I always keep my promises even if I have to do difficult or unpleasant things. | I sometimes break promises because they are difficult or unpleasant to fulfill. |
| Co4. When I start doing something important, I always finish it on time. | I often start doing things that I do not finish on time. |
| Co5. I make strong efforts to be excellent in my profession, or in my studies, in order to achieve great results. | I work just as much as necessary to keep my job or pass my exams. I don't like to work or study hard. |
| Co6. I like order. I always keep everything in its right place. | My things are often scattered. I do not make efforts to keep them in order. |

**Openness**

| O1. I like films and books about the art, history, and traditions of foreign countries that are very different from mine. | I am bored by films and books about the art, history, and traditions of foreign countries that are very different from mine. |
| --- | --- |
| O2. I love poetry. | I am bored by poetry. |
| O3. I am interested in sciences, such as medicine and psychology, explaining how the human body and mind work. | I am bored by sciences like medicine and psychology. |
| O4. I often try to find new solutions to old problems. | I prefer solutions that have stood the test of time. I see no point in looking for new ones. |
| O5. I would like to do new things each day. | I would like to have a life without much change. |
| O6. I like complex tasks that make me think hard. | I dislike tasks that make me think a lot. I prefer simple tasks. |
| O7. I like to have variety in my life: to do different things every day. | I want each day in my life to be like the one before. |
| O8. I like many different types of music | I like just one type of music or none at all. |

**Agreeableness**

| Ag1. I trust most people. | I mistrust most people. |
| --- | --- |
| Ag2. I do big favors for people just to see them happy. | I do big favors only for people who also do big favors for me. |
| Ag3. I find it easy to forgive people who have done something wrong to me. | I find it hard to forgive people who have done something wrong to me. |
| Ag4. I am nice to almost everybody. | I can be quite unpleasant or rude to people that I do not like. |
| Ag5. I am a very honest person. I would never do any harm to people by cheating. | I sometimes deceive people so that I get what I want from them. |
| Ag6. I avoid bragging. I don't want people to dislike me or envy me if I show off too much. | I like to show off even if people dislike me or envy me for that. |
| Ag7. I rarely criticize anybody | I often criticize people when I see that they have done something wrong. |
| A8. I easily agree with people, whatever we talk about. | I often argue with people and disagree about all kinds of things. |

**References**

Brosseau-Liard, P. E., Savalei, V., & Li, L. (2012). An investigation of the sample performance of two nonnormality corrections for RMSEA. *Multivariate Behavioral Research*, *47*(6), 904-930.

Brosseau-Liard, P. E., & Savalei, V. (2014). Adjusting incremental fit indices for nonnormality. *Multivariate Behavioral Research*, *49*(5), 460-470.

Brown, T. A. (2015). *Confirmatory factor analysis for applied research*. Guildford Press.

Browne, M. W., & Cudek, R. (1993). Alternate ways of assessing model fit. In K. A. Bollen & J. S. Long (Eds.), *Testing structural equation models* (pp. 136–162). Newbury Park, CA: Sage.

Davidov, E., Meuleman, B., Cieciuch, J., Schmidt, P., & Billiet, J. (2014). Measurement equivalence in cross-national research. *Annual review of sociology*, *40*, 55-75.

Fornell, C., & Larcker, D. F. (1981a). Evaluating structural equation models with unobservable variables and measurement error. *Journal of Marketing Research*, 18(1), 39-50. https://doi.org/10.2307/3151312

Hu, L. T., & Bentler, P. M. (1999). Cutoff criteria for fit indexes in covariance structure analysis: Conventional criteria versus new alternatives. *Structural equation modeling*, 6(1), 1-55.

Kline, R.B., 2015. Principles and Practice of Structural Equation Modeling. Guilford Press, New York, NY.

Putnick, D. L., & Bornstein, M. H. (2016). Measurement invariance conventions and reporting: The state of the art and future directions for psychological research. *Developmental review*, *41*, 71-90.

Rönkkö, M., & Cho, E. (2020). An updated guideline for assessing discriminant validity. *Organizational Research Methods*, 1094428120968614.

Rosseel, Y. (2012). Lavaan: An R package for structural equation modeling and more. Version 0.5–12 (BETA). *Journal of statistical software*, 48(2), 1-36.

Saris, W. E., Satorra, A., & Van der Veld, W. M. (2009). Testing structural equation models or detection of misspecifications?. *Structural Equation Modeling*, *16*(4), 561-582.

Sass, D. A., Schmitt, T. A., & Marsh, H. W. (2014). Evaluating model fit with ordered categorical data within a measurement invariance framework: A comparison of estimators. *Structural Equation Modeling*, 21(2), 167-180.

Satorra, A. (2000). Scaled and adjusted restricted tests in multi-sample analysis of moment structures. In Heijmans, R.D.H., Pollock, D.S.G. & Satorra, A. (eds.), *Innovations in multivariate statistical analysis*. A Festschrift for Heinz Neudecker (pp.233-247). London: Kluwer Academic Publishers.

Savalei, V. (2018). On the computation of the RMSEA and CFI from the mean-and-variance corrected test statistic with non-normal data in SEM. *Multivariate Behavioral Research*, *53*(3), 419-429.

Wu, H., & Estabrook, R. (2016). Identification of confirmatory factor analysis models of different levels of invariance for ordered categorical outcomes. *Psychometrika*, 81(4), 1014-1045.

Zumbo, B. D., Gadermann, A. M., & Zeisser, C. (2007). Ordinal versions of coefficients alpha and theta for Likert rating scales. *Journal of modern applied statistical methods*, *6*(1), 21-29.

1. It must be underscored that, as far as we know, there are no recommendations in the existing MI literature regarding the magnitude of the differences in alternative global fit indices between models assuming different levels of MI and using the Wu-Estabrook approach, in order to claim that one or other level of invariance is not a plausible assumption. Yet, insignificant values of the χ2 difference test (quite rare occasion in applied MI tests) seem to be quite convincing evidence in favor of the conclusion that the factor loadings in our Big Five personality model are more or less equivalent across various subgroups of the Mongolian sample. [↑](#footnote-ref-1)
2. There are no universally recognized decision rules regarding the magnitude of the differences in values of various global fit indices between models with different levels of invariance to reject the invariance assumption, when categorical indicator variables are used. We refer to Chen’s (2007) cutoff guidelines, as the most popular in applied research (and most often cited in influential review articles, e.g. Davidov et al. 2014) , although those guidelines are based on simulation experiments with continuous indicators. Generally, it seems that “one size does not fit all”: sensitivity of various fit indices to lack of measurement invariance depends on many factors, so any cutoff values should be used with great caution (Wass et al. 2014). [↑](#footnote-ref-2)
